# Prevalence of anxiety and depression in people with different types of cancer or haematologic malignancies: a cross-sectional study

**DOI:** 10.1017/S2045796022000592

**Published:** 2022-10-17

**Authors:** E. L. Zeilinger, C. Oppenauer, M. Knefel, V. Kantor, C. Schneckenreiter, S. Lubowitzki, K. Krammer, C. Popinger, A. Kitta, L. Kum, F. Adamidis, M. Unseld, E. K. Masel, T. Füreder, S. Zöchbauer-Müller, R. Bartsch, M. Raderer, G. Prager, M. T. Krauth, W. R. Sperr, E. Porpaczy, P. B. Staber, P. Valent, A. Gaiger

**Affiliations:** 1 Division of Hematology and Hemostaseology, Department of Internal Medicine I, Medical University of Vienna, Vienna, Austria; 2 Division of Palliative Medicine, Department of Internal Medicine I, Medical University of Vienna, Vienna, Austria; 3 Division of Clinical Psychology, Department of Psychology and Psychodynamics, Karl Landsteiner University of Health Sciences, Krems, Austria; 4 Department of Clinical and Health Psychology, Faculty of Psychology, University of Vienna, Vienna, Austria; 5 Division of Oncology, Department of Internal Medicine I, Medical University of Vienna, Vienna, Austria; 6 Ludwig Boltzmann Institute for Hematology and Oncology, Medical University of Vienna, Vienna, Austria

**Keywords:** Cancer, mental disorders, oncology, psycho-oncology

## Abstract

**Aims:**

Cancer patients often present with psychological symptoms that affect their quality of life, physical health outcomes and survival. Two of the most frequent psychiatric comorbidities are anxiety and depression. However, the prevalence of these disorders among cancer patients remains unclear, as studies frequently report varying rates. In the present study, we aimed to provide robust point estimates for the prevalence of anxiety and depression for both a mixed cancer sample and for 13 cancer types separately, considering confounding variables.

**Methods:**

In a sample of 7509 cancer outpatients (51.4% female), we used the *Hospital Anxiety and Depression Scale* to assess rates of anxiety and depression. Applying ordinal logistic regression models, we compared the prevalence of anxiety and depression between different cancer types, controlling for age and gender.

**Results:**

About one third of our sample showed symptoms of anxiety (35.2%) or depression (27.9%), and every sixth patient had a very likely psychiatric condition, with women being more frequently affected. Elderly patients more often showed signs of depression. The prevalence of anxiety and depression was significantly higher in lung and brain cancer patients, than in other cancer patients. Lowest depression rates were found in breast cancer patients.

**Conclusions:**

The prevalence of anxiety and depression is high in cancer patients. Type of cancer is an important predictor for anxiety and depressive symptoms, with lung and brain cancer patients being highly burdened. Considering a personalised medicine approach, physicians should take into account the high prevalence of psychiatric comorbidities and include psychiatric consultations in the treatment plan.

## Introduction

Cancer patients are more prone to psychiatric disorders than people without a chronic health condition (Niedzwiedz *et al*., 2019). Psychiatric symptoms negatively influence patients' quality of life, and also affect physical health outcomes and mortality (Unseld *et al*., 2021; Gaiger *et al*., 2022). Two of the most frequent psychiatric comorbidities are anxiety and depression (Pilevarzadeh *et al*., 2019; Unseld *et al*., 2019). Studies examining prevalence of anxiety and depression in cancer patients report widely varying estimates (Ng *et al*., 2011; Brandenbarg *et al*., 2019). Several aspects contribute to this wide variation in results including type of assessment and sample characteristics such as gender, age and cancer type (Krebber *et al*., 2014; Caruso *et al*., 2017; Niedzwiedz *et al*., 2019). Women report symptoms of psychiatric comorbidities more often (Massie, 2004; Walker *et al*., 2014; Wen *et al*., 2019; Salm *et al*., 2021), however, the effect of gender is not yet fully understood (Pitman *et al*., 2018). Another aspect contributing to the wide variability in reported prevalence estimates is the varying and sometimes even poor methodological quality of studies, for example in terms of sample size (Walker *et al*., 2014; Caruso *et al*., 2017). Furthermore, the cancer type can have an influence on the psychological burden of patients (Pitman *et al*., 2018). A large amount of studies include only one or a few types of cancer, such as breast or lung cancer, thus reflecting only a small part of the cancer patient population (e.g. Tsaras *et al*., 2018; Erim *et al*., 2019).

The aims of this study were to investigate the overall prevalence of anxiety and depression in cancer outpatients and to examine differences related to cancer type, age and gender. We provide robust point estimates for the prevalence of anxiety and depression for both a mixed cancer sample and for 13 cancer types separately, and identified which cancer types are most commonly associated with mental health problems.

## Method

### Materials

Anxiety and depression were assessed using the *Hospital Anxiety and Depression Scale* (HADS, Zigmond and Snaith, 1983). The HADS comprises 14-items, rated on a 4-point Likert scale with higher scores indicating more symptom-burden. Seven items each are summed up to arrive at the final scores for anxiety (HADS-A) and depression (HADS-D). These scores range from zero to 21. A score below eight indicates no anxiety/depression; a score between eight and ten indicates a possible anxiety/depressive condition, and scores above ten refer to significant anxiety/depression and thus a very likely psychiatric condition (Zigmond and Snaith, 1994; Snaith, 2003). The HADS has a stable two-factor structure in cancer outpatients (Zeilinger *et al*., 2022b). In the present sample, the HADS showed good internal consistencies (Cronbach's *α*), with *α* = 0.84 for HADS-A and *α* = 0.87 for HADS-D. A sociodemographic profile included the self-assessment of gender, age and previous psychiatric disorders (yes/no).

### Procedure

Participants comprised patients treated at the outpatient oncology and haematology clinic of the Medical University of Vienna in Austria. Inclusion criteria were: (1) confirmed diagnosis of cancer or other haematologic malignancies, (2) age ⩾ 18 years, (3) ability to give consent, (4) sufficient knowledge of German language. Informed consent was obtained from all participants. A response rate of 78 per cent was achieved. As common reasons for non-participation, the patients stated that they were not interested in participating in a study or that they did not have enough time. Collection of data was carried out in the years 2006–2022 as part of a standard psychosocial assessment at the research site. The study was conducted in accordance with the International Conference on Harmonisation E6 requirements for Good Clinical Practice outlined in the Declaration of Helsinki and the institutional ethics committee of the Medical University of Vienna granted approval (EC No.: 473/2006; 1241/2021).

### Statistical methods

We used percentages to report prevalence rates of anxiety and depression. To examine differences related to gender and previous psychiatric disorders (prior to the cancer diagnosis), *χ*^2^ tests were applied. The association of age with anxiety/depression was explored using Spearman's correlation coefficient. In order to compare the prevalence of anxiety/depression between different cancer types while controlling for age and gender, we used ordinal logistic regression models. Predictors did not show multicollinearity and the assumption of proportional odds was met. To assure robustness of results, only cancer types with *n* ⩾ 120 were included in regression analyses. Missing items in the HADS were imputed with the participant's subscale mean of the non-missing items if a maximum of two items were missing. This was the case for *n* = 103 participants. If more than two items were missing, the respective person was excluded from analysis. This was the case for *n* = 161 participants.

We did not interpret, nor report *p*-values, because these are highly dependent on sample size. With a sample as large as the present one, almost any test would result in a ‘significant’ *p*-value, without any practical meaning. Therefore, we report effect sizes, i.e. correlation coefficients, which are not dependent on sample size, and applied Cohen's guidelines (1988) for interpretation, with *r* ⩾ 0.1, *r* ⩾ 0.3, and *r* ⩾ 0.5 pertaining to a small, medium and large effect. In ordinal logistic regression analysis, statistically significant differences are assumed when the confidence intervals of the individual odds ratios do not overlap. *Strengthening the Reporting of Observational Studies in Epidemiology* guidelines were followed for reporting. Data analysed in this study is available in the OSF repository (Zeilinger and Gaiger, 2022).

## Results

### Patients

The study sample included *N* = 7509 (51.4% female) patients, with a mean age of 58.9 years (s.d. = 14) and an age range of 18–96 years. There were 1523 patients with a haematological diagnosis (cancer or other malignancies) and 5986 patients with a solid tumour. Gender and age distribution did not differ statistically between these two groups. The most frequent solid tumour diagnoses were breast cancer (*n* = 1194, 15.9%) and lung cancer (*n* = 1069, 14.2%). Table 1 depicts all cancer diagnoses. For 6372 patients, self-reported data on prior psychiatric disorders were available. Of these, 805 (10.7%) indicated that they had experienced a psychiatric disorder prior to their cancer diagnosis.

**Table 1.** Types of cancer in the sample

Cancer type	Total sample *N* = 7509		Women *n* = 3863		Men *n* = 3646	
	*n*	%	*n*	%	*n*	%
Haematological cancer / malignancies	1523	20.3	726	18.8	797	21.9
Solid tumour	5986	79.7	3137	81.2	2849	78.1
Breast	1194	15.9	1166	30.2	28	0.8
Lung	1069	14.2	480	12.4	589	16.2
Soft tissue	495	6.6	235	6.1	260	7.1
Pancreas	490	6.5	223	5.8	267	7.3
Colon/rectum	465	6.2	177	4.6	288	7.9
Head & neck	418	5.6	126	3.3	292	8.0
Brain	390	5.2	189	4.9	201	5.5
Stomach/oesophagus	302	4.0	85	2.2	217	6.0
Kidney/urinary tract/bladder	282	3.7	81	2.1	201	5.5
Female genital organs	231	3.1	231	6.0	–	–
Prostate	148	2.0	–	–	148	4.1
Testis	120	1.6	–	–	120	3.3
Hepatobiliary	114	1.5	39	1.0	75	2.1
Melanoma	87	1.2	29	0.8	58	1.6
Thyroid	82	1.1	37	1.0	45	1.2
Other solid	99	1.3	39	1.0	60	1.6

### Anxiety

Overall, 2642 (35.2%) patients experienced anxiety symptoms. Of these 1220 (16.2%) showed significant anxiety symptoms pertaining to a very likely anxiety condition. There was only a weak relation between age and anxiety (*r_sp_* =   −0.1), with younger patients showing slightly more anxiety symptoms. Women had significantly higher rates of anxiety (20.6%) than men (11.7%), with a small statistical effect (*χ*^2^(2, *N* = 7509) = 162.66, *r* = 0.15). A psychiatric disorder prior to the cancer diagnosis had only a small effect on the presence of anxiety (*χ*^2^(2, *N* = 6372) = 336.4, *r* = 0.23).

**Fig. 1.**
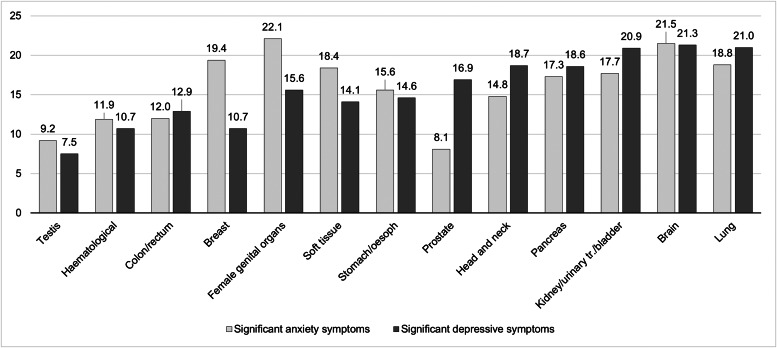
Prevalence of significant anxiety and depressive symptoms according to cancer type. The figure shows the proportion of patients with significant anxiety or depressive symptoms, as measured by the HADS with a respective score higher than 10. The y-axis represents percentages. oesoph, oesophagus; urinary tr., urinary tract.

**Fig. 2.**
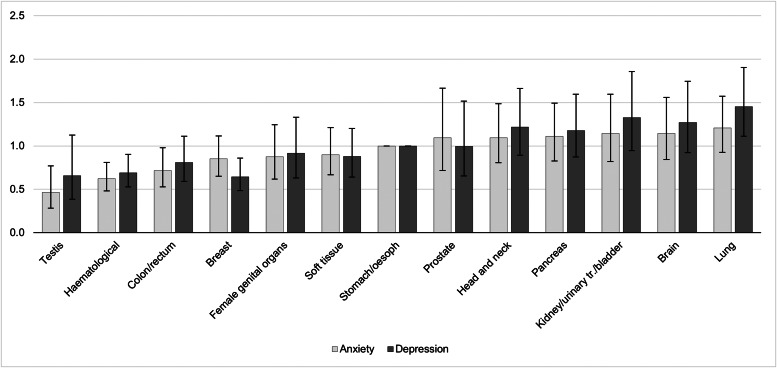
Odds ratios and confidence intervals of anxiety and depression according to cancer types. The figure shows the results of linear logistic regression analysis, controlling for age and gender. The bars represent the odds ratios (OR) for each cancer type including confidence intervals. Stomach/oesophagus cancer was the reference category with OR  =  1 and no computable confidence interval. A significant difference in anxiety/depressive symptoms between cancer types exists when the confidence intervals do not overlap, e.g. the prevalence of anxiety is significantly higher in patients with lung cancer compared to patients with testis or haematologic malignancies. oesoph, oesophagus; urinary tr., urinary tract.

The prevalence of anxiety according to different cancer types are detailed in Fig. 1 and Table 2. Cancer types with high raw scores in anxiety were cancer of the female genital organs, breast cancer, lung cancer and brain cancer. However, these percentages are not controlled for age and gender. By using an ordinal regression analysis, we compared anxiety rates between cancer types without a gender bias. The results (detailed in Table 3 and plotted in Fig. 2) still indicate a high prevalence of anxiety in lung and brain cancer, but not in cancer of the female genital organs or breast cancer. Cancer types with lowest anxiety symptoms were testis, haematologic and colon/rectum cancer. Patients with testis cancer had significantly less anxiety symptoms than patients with lung, brain, kidney/urinary tract/bladder, pancreas and head and neck cancer. Patients with haematologic malignancies had significantly less anxiety symptoms than patients with lung, brain, kidney/urinary tract and pancreatic cancer. All other cancer types did not differ from each other significantly.

**Table 2.** Prevalence of anxiety and depression in patients with different cancer types according to the *Hospital Anxiety and Depression Scale*

Cancer type	Anxiety						Depression					
	No anxiety		Possible anxiety		Significant anxiety		No depression		Possible depression		Significant depression	
	*n*	%	*n*	%	*n*	%	*n*	%	*n*	%	*n*	%
Haematological	1110	72.9	232	15.2	181	11.9	1166	76.6	194	12.7	163	10.7
Breast	709	59.4	253	21.2	232	19.4	909	76.1	157	13.1	128	10.7
Lung	633	59.2	235	22.0	201	18.8	643	60.1	202	18.9	224	21.0
Soft tissue	321	64.8	83	16.8	91	18.4	363	73.3	62	12.5	70	14.1
Pancreas	302	61.6	103	21.0	85	17.3	315	64.3	84	17.1	91	18.6
Colon/rectum	333	71.6	76	16.3	56	12.0	344	74.0	61	13.1	60	12.9
Head & neck	262	62.7	94	22.5	62	14.8	274	65.6	66	15.8	78	18.7
Brain	231	59.2	75	19.2	84	21.5	260	66.7	47	12.1	83	21.3
Stomach/oesophagus	200	66.2	55	18.2	47	15.6	211	69.9	47	15.6	44	14.6
Kidney/urinary tract/bladder	182	64.5	50	17.7	50	17.7	179	63.5	44	15.6	59	20.9
Female genital organs	135	58.4	45	19.5	51	22.1	159	68.8	36	15.6	36	15.6
Prostate	101	68.2	35	23.6	12	8.1	103	69.6	20	13.5	25	16.9
Testis	95	79.2	14	11.7	11	9.2	99	82.5	12	10.0	9	7.5
Hepatobiliary	69	60.5	29	25.4	16	14.0	74	64.9	26	22.8	14	12.3
Melanoma	64	73.6	16	18.4	7	8.0	53	60.9	21	24.1	13	14.9
Thyroid	50	61.0	13	15.9	19	23.2	56	68.3	12	14.6	14	17.1

**Table 3.** Results of ordinal logistic regression analysis comparing anxiety and depression prevalence between different cancer types

Cancer type	Anxiety					Depression				
	estimate	s.e.	OR	Lower limit	Upper limit	estimate	s.e.	OR	Lower limit	Upper limit
Haematological	−0.472	0.134	0.624	0.480	0.810	−0.372	0.138	0.689	0.526	0.903
Breast	−0.162	0.138	0.851	0.649	1.115	−0.440	0.146	0.644	0.484	0.858
Lung	0.188	0.135	1.207	0.927	1.572	0.375	0.138	1.454	1.110	1.905
Soft tissue	−0.107	0.152	0.898	0.667	1.210	−0.130	0.160	0.878	0.642	1.200
Pancreas	0.105	0.151	1.111	0.827	1.493	0.165	0.154	1.179	0.871	1.596
Colon/rectum	−0.331	0.157	0.718	0.528	0.978	−0.211	0.162	0.810	0.590	1.112
Head and neck	0.090	0.156	1.095	0.807	1.485	0.196	0.159	1.216	0.891	1.661
Brain	0.136	0.157	1.145	0.842	1.558	0.239	0.163	1.270	0.923	1.746
Stomach/oesophagus^a^	0	–	1	–	–	0	–	1	–	–
Kidney/urinary tract/bladder	0.134	0.171	1.143	0.818	1.596	0.282	0.172	1.326	0.947	1.857
Female genital organs	−0.133	0.179	0.875	0.616	1.243	−0.088	0.191	0.915	0.630	1.331
Prostate	0.089	0.215	1.094	0.718	1.666	−0.006	0.215	0.994	0.652	1.516
Testis	−0.763	0.257	0.466	0.282	0.771	−0.419	0.274	0.658	0.385	1.125

*Note.* s.e., standard error; OR: odds ratio. Model controlled for gender and age.

a Stomach/oesophagus cancer was used as reference category with estimate set to 0 and odds ratio set to 1. No standard error or limits can be computed for the reference category.

### Depression

Overall, there were 2229 (29.7%) patients experiencing depressive symptoms. Of these, 1122 (15%) showed significant depressive symptoms, strongly indicating a depressive condition. Age showed only a weak relation to anxiety symptoms (*r_sp_* =   0.11), with older patients having slightly more symptoms of depression. There were no gender differences (*χ*^2^(2, *N* = 7509) = 3.05, *r* = 0.02). A total of 15.6% of women and 14.2% of men reported significant depressive symptoms. A psychiatric disorder prior to the cancer diagnosis had a small effect on the presence of depression (*χ*^2^(2, *N* = 6372) = 199.11, *r* = 0.18).

The prevalence of depression according to different cancer types are detailed in Fig. 1 and Table 2. Table 3 and Fig. 2 depict the results of ordinal regression analysis. Cancer types with highest depressive symptoms were lung, kidney/urinary tract/bladder and brain cancer. The lowest rate of depressive symptoms was found in breast cancer, testis cancer and haematologic malignancies. Patients with breast cancer had significantly fewer symptoms of depression than patients with lung cancer, kidney/urinary tract/bladder cancer, brain cancer, head and neck cancer and pancreatic cancer. All other cancer types did not differ significantly from each other in relation to depressive symptoms.

## Discussion

The present study examined prevalence rates of anxiety and depression in a large sample of cancer outpatients with different cancer diagnoses. We found about 35 per cent of cancer patients with symptoms of anxiety, and 30 per cent with symptoms of depression. Significant symptoms, strongly indicating a psychiatric condition, were present in 16.2 per cent for anxiety and 15 per cent for depression, as compared to around four to six per cent globally and in the German population (Jacobi *et al*., 2004; Baxter *et al*., 2014). The prior presence of a psychiatric disorder before cancer diagnosis had only a small effect on the current presence of anxiety and depression. A cancer diagnosis can therefore be regarded as a decisive factor for the development of a psychiatric condition both in the sense of an adjustment reaction and a biopsychosocial response to the disease.

In our study, the prevalence of anxiety was higher in younger patients. This can have several reasons, including that younger patients are less experienced and prepared to cope with physical limitations and to emotionally and cognitively accept their illness; an that the daily routine of younger patients is often more interrupted by a cancer diagnosis (Linden *et al*., 2012). We also found that women were more likely to show symptoms of anxiety, which has also been well documented in previous studies (Linden *et al*., 2012; Salm *et al*., 2021). This may not necessarily be linked to a higher anxiety prevalence, but can also be caused by women being more open in communicating their symptoms, and by the tendency to use emotional approach coping (Linden *et al*., 2012; Salm *et al*., 2021). A study with long-term cancer survivors compared anxiety rates to a matched reference population and found that – compared to their respective healthy reference group – men had relatively more symptoms of anxiety than women (Oertelt-Prigione *et al*., 2021). This also must be regarded with caution and should not lead to the conclusion that male cancer survivors are more burdened. Women generally show higher anxiety levels where a small increase can have a clinically significant meaning, whereas a possible larger net increase in a lower anxiety range may be clinically less relevant.

Controlling for gender is crucial in examining predictors for anxiety. The raw scores of our data indicate that cancer of the female genital organs and breast cancer were among the cancers most frequently affected by anxiety. However, controlling for gender in a regression model revealed that these cancers, which exclusively or primarily affect women, are not themselves indicators of high anxiety scores, but that the observed high anxiety prevalence is attributable to gender.

The prevalence of depression was independent of gender, but we observed a small effect of age, with elderly people having depressive symptoms more frequently. This may be due to limited abilities to ask for help and to communicate with others or worries related to treatment costs and family financial difficulties (Hong and Tian, 2014). Elderly people have to cope with general functional impairments and the loss of loved ones, and have often a poorer network of social support than younger patients (Polikandrioti *et al*., 2008). However, age as a risk factor for depression remains controversial, as there are also studies indicating a reverse effect with younger patients showing signs of depression more often (Walker *et al*., 2014; Peng *et al*., 2019).

Our data demonstrates that there are significant differences in psychological burden between cancer types, independent of gender and age. These differences can be caused by different prognosis, pain levels, the degree of body image disruption and tumour- or treatment-related neuropsychiatric (side) effects (Pitman *et al*., 2018). In the present study, we found high anxiety and depression rates in patients with lung and brain cancer. Both cancer types have a high mortality rate, which can promote fear or contribute to depression (Hong and Tian, 2014; Polanski *et al*., 2016). Small cell lung cancer can also provoke hormonal effects, e.g. syndrome of inappropriate antidiuretic hormone secretion, which may lead to depression (Pitman *et al*., 2018). Symptoms commonly associated with these two cancer types, such as difficulty breathing or loss of control due to central nervous system damage, can be both life-threatening and particularly distressing, further contributing to increased psychological symptom burden. We found that breast cancer patients had a low rate of depression, after controlling for gender. Breast cancer patients receive a comparably high amount of support with multiple support groups and awareness raising activities being in place. This may facilitate a lower rate of psychiatric comorbidities.

International data illustrate that only a small part of cancer patients with psychiatric symptoms receive appropriate treatment (Walker *et al*., 2014; Naser *et al*., 2021). This aspect may even have been exacerbated by the COVID-19 pandemic. Particularly vulnerable populations such as cancer patients with low socioeconomic status showed low treatment-seeking behaviour during the pandemic (Zeilinger *et al*., 2022a). Given the generally high proportion of psychologically burdened cancer patients, low-threshold mental health treatment should be an integral part of interdisciplinary cancer care.

### Strength and limitations

This study is limited by some factors. First, we did not control for cancer stage, treatment phase, including current or past chemotherapy or therapy response. However, using this unselected mixed cancer patient sample, we can report prevalence rates as would actually be encountered in an outpatient clinic and thus provide a realistic picture of the actual burden of outpatient cancer patients. Second, we used a screening instrument to assess anxiety and depression. Screening instruments are often over inclusive regarding a potential diagnosis. We tried to mitigate this aspect by using two cut-offs in our analysis; one to indicate the proportion of patients experiencing anxiety or depressive symptoms; and one higher cut-off that represents a very likely psychiatric condition. It should also be noted that symptoms of depression and anxiety can be part of other psychiatric conditions, such as post-traumatic stress disorder, bipolar disorder or adjustment disorder. Furthermore, the HADS has not yet been validated among brain cancer patients. It remains unclear to what extent pathological factors related to the disease, including alterations in perception or neuroanatomy, may influence self-report of psychiatric symptoms. Especially in patients with brain cancer, high symptom burden may also be related to organic mental disorders. As a single-centre study, the generalisability of results may be limited. The large sample size of the present study should be emphasised as a strength. This large sample size can statistically reduce potential sampling bias in the data and provides the opportunity for reliable prevalence estimates.

## Conclusions

Given the generally high proportion of psychologically burdened cancer patients, mental health treatment should be an integral part of interdisciplinary cancer care. The type of cancer is an important predictor for anxiety and depressive symptoms, with lung and brain cancer patients being highly burdened. Mental health treatment options should therefore be enforced with an emphasis on low-threshold offers and prevention activities following the well elaborated support and intervention strategies for breast cancer patients. A stepped care approach could help ensure that patients who need mental health support receive it while conserving staff resources. Physicians as well as mental health professionals may consider the high prevalence of psychiatric comorbidities, especially in lung and brain cancer patients, and monitor for patient reported outcomes related to anxiety and depression to ensure treatment according to the principles of personalised medicine.

## Data Availability

The data that support the findings of this study are openly available in OSF at https://doi.org/10.17605/OSF.IO/7THFY
